# Clinical study on vacuum assisted closure combined with multiple flaps in the treatment of severe hand trauma

**DOI:** 10.12669/pjms.38.1.4631

**Published:** 2022

**Authors:** Quan Wang, Xu Zhang, Wentao Sun, Hua Li

**Affiliations:** 1 Quan Wang, Department of Hand and foot surgery, Harrison International Peace Hospital, Hebei, Hengshui 053000, China; 2 Xu Zhang, Department of Orthopedics, Orthopedic Hospital of Sichuan Province, Chengdu, Sichuan 610041, China; 3 Wentao Sun, Department of Hand and foot surgery, Harrison International Peace Hospital, Hebei, Hengshui 053000, China; 4 Hua Li, Department of Hand and foot surgery, Harrison International Peace Hospital, Hebei, Hengshui 053000, China

**Keywords:** Flap treatment, Vacuum assisted closure, Severe hand trauma, Survival rate

## Abstract

**Objectives:**

To investigate the effect and clinical value of the application of vacuum assisted closure (VAC) combined with multiple flaps in the treatment of severe hand trauma.

**Methods:**

A total of 100 patients with severe hand trauma admitted to Harrison International Peace Hospital from September 2015 to September 2020 were selected and randomly divided into two groups according to the randomized block method: the single flap repair group and the combined repair group, with 50 patients in each group. Patients in the single flap repair group underwent flap repair according to their condition, while those in the combined repair group were treated with VAC prior to flap repair. The range of motion and hand sensation scores were compared between the two groups, and their levels of interleukin-8 (IL-8), tumor necrosis factor (TNF) and lipopolysaccharide (LPS) were tested by enzyme-linked immunosorbent assay (ELIS). Moreover, the flap survival rate and the incidence of adverse events were recorded and compared between the two groups.

**Results:**

Compared with the single flap repair group, the combined repair group had higher range of motion and hand sensation score (p<0.05), lower levels of IL-8, TNF and LPS (p<0.05), higher flap survival rate (p<0.05), and lower incidence of adverse events (p<0.05).

**Conclusion:**

VAC combined with multiple flaps boasts significant trauma repair effect and preferable clinical application value in the treatment of patients with severe hand trauma, which is principally reflected in significantly improving the hand function of patients and remarkably alleviating the inflammatory response of patients.

## INTRODUCTION

The vital organs that people use in life and work are inextricably linked to the application of hands. Hand trauma is a frequent injury in life because humans need their hands for productive activities and life all the time.^1^,^2^ Patients suffering from bone and joint injuries, such as severe hand open skin injuries, tendon injuries, nerve and blood vessel injuries, must undergo surgery in time, and such patients are called patients with severe hand trauma.^3^ When a patient suffers from hand trauma, different fingers or even multiple fingers are often involved. Difficulty in wound healing can be attributed to a variety of reasons, mainly including large tissue defects, vascular exposure and poor blood supply.^4^ Wounds are difficult to heal by traditional means, and flap treatment, by contrast, has become the preferred treatment for clinical patients due to its multiple advantages.^5^ With the help of VAC therapy, negative pressure treatment is carried out on the wound to reduce the bacteria on the wound surface, thus promoting wound healing. Meanwhile, patent dressings are placed on the wound site, which is conducive to the drawing of the wound edge and the formation of cell-level granulation tissue.^6^ In this paper, vacuum assisted closure (VAC) combined with multiple flaps was applied to explore its therapeutic effect on patients with severe hand trauma, so as to provide reference value for its clinical application.

## METHODS

A total of 100 patients with severe hand trauma admitted to Harrison International Peace Hospital from September 2015 to September 2020 were selected and randomly divided into two groups according to the randomized block method: the single flap repair group and the combined repair group, with 50 patients in each group. In the single flap repair group, there were 23 female patients and 27 male patients, aged 24 - 41 years, with an average age of 33.2±5.2 years. In the combined repair group, there were 21 female patients and 29 male patients, aged 21 - 39 years old, with an average age of 32.2±4.9 years. No statistical difference could be observed in the general information of the two groups of patients, which were comparable.

### Ethical Approval:

The study was approved by the Institutional Ethics Committee of Harrison International Peace Hospital on June 5, 2018 (No.:2018-2-003), and written informed consent was obtained from all participants.

### Selection criteria:

Patients with severe hand trauma confirmed by clinically relevant examination and imaging examination and evaluation in our hospital based on their medical history.

### Exclusion criteria:

- Patients with incomplete data;
- Patients complicated with cardiovascular and cerebrovascular diseases;
- Patients with mental disorders;
- Patients who have recently undergone related surgery;
- Patients with malignant tumors.

***Flap preparation:*** Reverse-flow Island flap of supracarpal cutaneous branch of ulnar artery: By setting the position 4cm from the pisiform bone as the rotation point, and the line between the pisiform bone and the internal epicondylar of the humerus as the axis line, the wound design was carried out based on the size of the flap, and the supracarpal cutaneous branch of the ulnar artery was exposed under the ulnar flexor carpi tendon. The cutaneous vessels and fascial pedicle were protected at all times, and the flap was separated from deep fascia of the forearm.

Fascial pedicle island flap with superficial branch of radial nerve: By setting the center of the snuff socket as the rotation point, and the connection between the rotation point and the middle and outer thirds of the elbow stripes as the axis line, the wound design was carried out based on the size of the flap and the length of the pediclethe. The flap was separated and cut below deep fascia, the superficial branch of the radial nerve was found and cut proximally, and the fascial pedicle was protected at all times to separate to the snuff nest.

Reverse-flow Island flap of dorsal interosseous artery of forearm: By setting the position around 2.5cm of the ulnar styloid process as the rotation point, and the lower two-thirds of the line connecting the lateral epicondyle of the humerus to the radial edge of the ulnar microcephalus as the axis line, the wound design was carried out based on the length of the pedicle and the size of the flap. At the same time, the flap was separated under the deep fascia during the incision, and the interosseous dorsal artery was thoroughly dissected proximally through the ulnar extensor carpi tendon and extensor little finger muscle. There are usually one or two large cutaneous branches at the lower edge of the rotator muscle. The vascular fascial pedicle was protected at all times, and the interosseous dorsal artery was cut off near the cutaneous vessels.

Anterolateral thigh flap: At the position of the midpoint of the connection between the anterior superior iliac spine and the lateral edge of the patella, the lateral femoral circumflex artery was examined by a pen-type vascular Doppler detector, and then the descending branch of the first perforating artery was marked. Wound design was performed based on the length of the pedicle and the size of the flap. The thigh was separated from the rectus muscle and the lateral muscle space outside the thigh, and the descending branch of the lateral femoral circumflex blood vessel was exposed. After that, the free blood vessel was carefully dissected, and the cutaneous branch blood vessels were protected. Near midline, the fascia lata was cut and separated to depth. At the same time, a small part of the muscle tissue was cut to enclose the blood vessels in the muscle sleeve.

### VAS Therapy:

In this study, all patients were first subjected to local or general anesthesia after entering the operating room. After anesthesia, the patients’ wounds were debrided, and the necrotic tissues were cleaned up. The injured sites were sequentiously soaked by hydrogen peroxide and iodophor, and then placed under the VAC device. Vacuum aspiration was set by the physician as an intermittent aspiration with a pressure of 10 to 12kPa. Patients were subjected to vacuum aspiration for one week to observe the growth of the granulation on the wound until the new bud grew and the VAC was discontinued. After that, the patients were subject to subsequent surgery, and the wounds were covered with dressing after debridement to facilitate the cleaning of the wounds.

### Surgical Methods:

Metacarpal and phalangeal fractures were fixed with steel plates, and the tendons were repaired with non-invasive sutures. The design flap was selected according to the severity of the patient’s condition and the area of injury. After the flap was removed, the free anterolateral femoral flap was directly placed in the wound area. The artery of the flap matched the distal radial artery, the veins matched the superficial veins of the forearm, and the skin was sutured. Revirse-flow island skin flap of the cutaneous branch of the ulnar artery was moved to the wound area via the open channel. Important veins were close to the uninjured superficial veins of the hand, and the skin was sutured. The fascial pedicle island flap with superficial branch of radial nerve and the acute island flap of the dorsal interosseous artery of the forearm were transferred to the wound area via the subcutaneous tunnel, and the skin was sutured.

### Range of motion and hand sensation score:

The range of motion of the two groups of patients was measured with a protractor before and after the repair. A two-point discrimination test was performed on the two groups of patients, and then the hand sensation of the patients was evaluated, including the distance between any two points of the hand of <4 mm, four to six mm, 7 to 10 mm, and <10 mm. The smaller the distance between the two points of discrimination, the higher the score, indicating the better the recovery of hand trauma.

### Levels of IL-8, TNF and LPS:

Before and after the repair, 3 ml of fasting venous blood was drawn from the patients in the two groups in the morning for testing, and changes in their levels of IL-8, TNF and LPS were detected by enzyme-linked immunosorbent assay. 50 mm carbonate buffer was added to the reaction well of polystyrene to dilute the venous blood, followed by antigen. After capping, the obtained diluent was stored in a refrigerator at a constant temperature of 4 °C for 24 h. The diluent was washed three times on the second day and dried. The test samples were diluted with 0.02 mol /L Tris-HCl (pH 7.4) buffer diluent, and then placed in each well. Positive and negative control specimens were placed in the wells and placed at 42 °C for 60 min, then the obtained specimens were stored. After removing the liquid in the wells, the wells were washed three times and dried. 0.1 mL of IL-8, TNF and LPS antibodies were placed in each well, kept for 60 min, and then the liquid was removed. The wells were washed three times and then dried. The substrate solution (0.1 mol /L Na2HPO4, 0.05 mol /L citric acid) was put into each well, and 0.1 ml o-phenylenediamine was added after uniformity, shaded for 20 min, and then two mol/L H2SO4 (0.05 ml) was put into the well, and the reaction was terminated. The levels of IL-8, TNF and LPS were analyzed by measuring the A450 value with a microplate reader.

### Flap survival Rate:

The patient’s flap survival rate was judged according to the four levels: excellent, good, fair, and poor. Excellent means that the patients recovered to normal level after repair; Good means that the patients’ flap survived well, the blood supply status of the injured hand was good, and the hand function was significantly recovered; Fair indicates that a small part of the patients’ flaps were necrotic, the hand function was slightly restored, and the blood supply status was improved. Poor indicates no change in hand function and complete necrosis of the flap.

### Adverse Events:

The adverse events of the two groups, including marginal necrosis, infection, liquefaction and anemia, were recorded and compared by senior physicians in our hospital.

### Statistical Processing:

All data in this study were analyzed and processed by SPSS21.0 software, and the counting data were expressed represented by %. Intergroup comparison was performed by *x^2^* test, the measurement data were expressed as (x ¯ ±s), and intergroup comparison was tested by LSD-t. P<0.05 indicates a statistically significant difference.

## RESULTS

As shown in Table-I, before repair, the range of motion and hand sensation scores of the two groups were compared, with no statistical difference (p>0.05). After repair, the range of motion and hand sensation scores of the combined repair group were higher than that of the single flap repair group, with statistical differences (p<0.05).

**Table-I:** Comparison of range of motion and hand sensation score between the two groups before and after surgery (x̄±S).

Group	Number of cases (n)	range of motion		Hand sensation score	

		Before repair	After repair	Before repair	After repair
Single flap repair group	50	24.98±2.35	34.63±3.31	1.28±0.13	3.06±0.26
Combined repair group	50	25.25±2.39	43.57±5.24	1.26±0.11	4.79±0.51
t		0.570	10.200	0.830	21.370
p		0.570	0.001	0.408	0.001

Before repair, the levels of IL-8, TNF and LPS of the two groups were compared, with no statistical difference (p>0.05); After repair, the levels of IL-8, TNF and LPS in the combined repair group were lower than those in the single flap repair group, with statistical differences (p<0.05). Table-II

**Table-II:** Comparison of levels of IL-8, TNF and LPS before and after surgery between the two groups (x̄±S).

Group	IL-8 (pg/mL)		TNF (pg/mL)		LPS (pg/mL)	

	Before repair	After repair	Before repair	After repair	Before repair	After repair
Single flap repair group	1.39±0.25	1.16±0.12	1.57±0.37	1.05±0.13	1.41±0.09	0.97±0.07
Combined repair group	1.36±0.26	0.67±0.09	1.59±0.36	0.56±0.46	1.43±0.11	0.52±0.05
t	0.588	23.100	0.274	7.248	0.995	36.990
p	0.558	0.001	0.785	0.001	0.322	0.001

The flap survival rates of the two groups of patients after surgery were compared. The flap survival rate of the combined repair group was higher than that of the single flap repair group, with a statistical difference (p<0.05). Table-III

**Table-III:** Comparison of flap survival rates between the two groups after surgery [N,%].

Group	Excellent	Good	Fair	Poor	Survival rate
Single flap repair group	15	14	12	7	41 (82.00)
Combined repair group	18	15	14	1	47 (94.00)
X^2^					3.409
p					0.065

The incidence of adverse events between the two groups was compared. The incidence of adverse events in the combined repair group was lower than that in the single flap repair group, with a statistically significant difference (p<0.05).Table-IV

**Table-IV:** Comparison of the incidence of adverse events between the two groups [N,%].

Group	Edge necrosis	Infection	Liquefaction	Anemia	Incidence of adverse events
Single flap repair group	2	1	3	2	8 (16.00)
Combined repair group	0	1	1	0	2 (4.00)
X^2^					4.000
p					0.045

## DISCUSSION

Severe hand trauma, which can be attributed to cutting, extrusion, twisting injuries, etc., mostly makes inroads on migrant workers and workers. Most migrant workers suffer from hand trauma owing to unskilled operation and mistakes of corn peeling machine.^7^ On the other hand, the condition of a few patients was aggravated due to a variety of reasons, such as the limited medical conditions in the local hospital where the patients were injured and the failure to treat the injury in a timely and correct manner due to the patient’s mishandling of the injury, thus affecting the treatment and prognosis.^8^ The rate of wound healing is twice as much as that of simple healing.^9^ Patients with hand trauma are often accompanied by multiple finger injuries, which is also a crucial consequence of the causes of hand and hand structure injuries^10^ In case of danger, five fingers cannot be separated in time, and hand injuries will be caused in most cases. Among the five fingers, the index finger and middle finger have a higher injury rate, followed by the ring finger and thumb, and finally the little finger.^11^ Hand trauma has a severe impact on the quality of life and family status of patients.^12^ Different parts of the wound have different properties, so skin flaps consistent with the wound can be selected for repair, which has a significant impact on the appearance of the wound, hand function and daily life of the patient.^13^ When it comes to refractory wounds, a variety of repair methods can be applied.^14^ With flap therapy, important tissues in the deep part of the wound can be covered and effectively treated, so that the ideal therapeutic effect can be realized on the appearance of the injured wound and the function of the limb.^15^ In the wake of the development of microsurgery technology in China, free flap transplantation stands out among numerous therapies with its own advantages, and many clinical problems have been solved by virtue of this method.^16^ VAC, as a medical polymer composite material composed of polyvinyl alcohol and gelatin sponge, has become a new technology for the treatment of hand trauma with its characteristics of covering soft tissues and repairing wounds. Medical polymer composite material can be used as the middle between the drainage tube and the drainage surface to connect the drainage point to the drainage surface and open the closed wound consistent with the wound surface, so as to prevent contamination of the wound and infection of complications.^17^ High vacuum aspiration boasts an obvious effect on the removal of infected wound surface in the early stage of wound repair. Specifically, with such a treatment, the exudate from the wound can be completely removed, the wound cleanness can be guaranteed, drainage can be complete to avoid the accumulation of exudate in the deep wound. In this way, the swelling of the tissue can be accelerated, effective local circulation can be promoted, healthy granulation tissue can be rapidly grown, and tissue repair can be accelerated.^18^

Most of the patients with hand trauma need to undergo hand surgery, which will cause psychological and physiological effects on the patients, as well as anxiety, depression and other adverse events to a large extent.^19^ The sensory function of hands has a great influence on human daily life, work, and even physical and mental health.^20^ A certain degree of sensory impairment can be caused by median nerve injury, in which sensory impairment is more obvious in the middle finger, thumb, and distance of the middle finger. Symptoms such as an inability to rotate the arm, an inability to bend the thumb and a decline in wrist flexion were classified as dyspexia. Despite the fact that hand nerve injuries can be repaired by surgery, a longer period of sensory function recovery is needed, and most patients will remain with sensory dysfunction.^21^ As shown in the results of this study, patients with severe hand trauma treated with VAC combined with multiple flaps have higher range of motion and hand sensation scores, indicating that the application of VAC combined with multiple flaps boasts the efficacy of significantly improving the hand function of patients.

It has been proved in related studies that in case of skin wounds, inflammatory factors are obviously expressed, and the wound is difficult to heal, which has a close bearing on the high expression of inflammation over a long period of time. The levels of IL-8, TNF and LPS are all important inflammatory factors, which are obviously expressed in surgical trauma. Relevant studies have shown that VAC technology features various advantages, such as promoting drainage of wound exudate and reducing bacterial culture medium in the wound, thus improving inflammatory response of patients on the wound.^22^ As shown in the results of this study, patients with severe hand trauma treated with VAC combined with multiple flaps have lower levels of IL-8, TNF and LPS, indicating that the application of VAC combined with multiple flaps can significantly reduce the inflammatory response of patients.

### Limitations of the study:

Nevertheless, shortcomings can still be seen in this study: the insufficient number of cases leads to a certain bias between the results and the actual situation. In view of this, proactive countermeasures will be taken in order to collect sufficient samples and increase the scope of the study, so as to obtain more accurate study results.

## CONCLUSIONS

With the vacuum aspiration method, muscle flaps with rich blood supply, muscle skin and tissue flaps containing well-known arteries can be repaired, the wound can be covered and at the same time, the local blood circulation nutrition status can be improved, and the ability to fight infection and heal can be enhanced. Specifically, the skin is firmly fixed to the wound, and the wound is continuously drained through the opening of the skin, which ensures the perfect attachment of the skin graft to the wound, and plays a vital role in wound healing.^23^ In this paper, patients with severe hand trauma who are treated with VAC combined with multiple flaps have a higher survival rate than single flap treatment, indicating that VAC combined with multiple flaps boasts a significant damage repair effect.

### Authors’ Contributions:

**QW & XZ:** Designed this study and prepared this manuscript, and are responsible and accountable for the accuracy or integrity of the work.

**WS:** Collected and analyzed clinical data.

**Hua Li** significantly revised this manuscript.
